# Supplementary material for: Splice-Junction-Based Mapping of Alternative Isoforms in the Human Proteome
Source: Cell Rep. Author manuscript; Available in PMC 2020 Jan 15. (PMC6961840; doi:10.1016/j.celrep.2019.11.026)
Supplement: 2 [file NIHMS1546469-supplement-2.zip › DF1/PXD010154/Testis/Testis_53_DCUN1D4_NSEILWITFQLN.pdf]

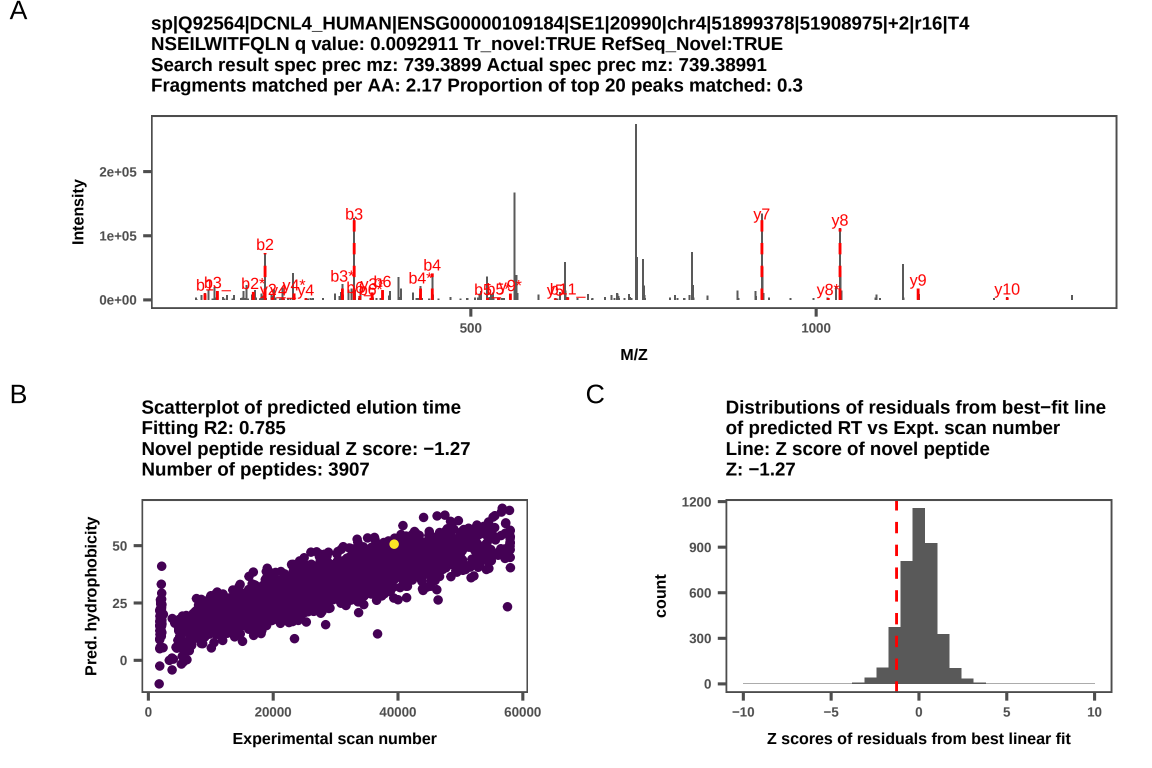
A
sp|Q92564|DCNL4_HUMAN|ENSG00000109184|SE1|20990|chr4|51899378|51908975|+2|r16|T4
NSEILWITFQLN q value: 0.0092911 Tr_novel:TRUE RefSeq_Novel:TRUE
Search result spec prec mz: 739.3899 Actual spec prec mz: 739.38991
Fragments matched per AA: 2.17 Proportion of top 20 peaks matched: 0.3
Intensity
2e+05
1e+05
0e+00
b2
b3
b3*
b4
b4*
y7
y8
y8*
y9
y10
500
1000
M/Z
B
Scatterplot of predicted elution time
Fitting R2: 0.785
Novel peptide residual Z score: −1.27
Number of peptides: 3907
Pred. hydrophobicity
50
25
0
0
20000
40000
60000
Experimental scan number
C
Distributions of residuals from best−fit line
of predicted RT vs Expt. scan number
Line: Z score of novel peptide
Z: −1.27
count
1200
900
600
300
0
−10
−5
0
5
10
Z scores of residuals from best linear fit
